# *TBX1* Mutation Identified by Exome Sequencing in a Japanese Family with 22q11.2 Deletion Syndrome-Like Craniofacial Features and Hypocalcemia

**DOI:** 10.1371/journal.pone.0091598

**Published:** 2014-03-17

**Authors:** Tsutomu Ogata, Tetsuya Niihori, Noriko Tanaka, Masahiko Kawai, Takeshi Nagashima, Ryo Funayama, Keiko Nakayama, Shinichi Nakashima, Fumiko Kato, Maki Fukami, Yoko Aoki, Yoichi Matsubara

**Affiliations:** 1 Department of Pediatrics, Hamamatsu University School of Medicine, Hamamatsu, Japan; 2 Department of Medical Genetics, Tohoku University School of Medicine, Sendai, Japan; 3 Department of Pediatrics, Kurashiki Central Hospital, Kurashiki, Japan; 4 Department of Pediatrics, Kyoto University School of Medicine, Kyoto, Japan; 5 Division of Cell Proliferation, United Centers for Advanced Research and Translational Medicine, Tohoku University Graduate School of Medicine, Sendai, Japan; 6 National Research Institute for Child Health and Development, Tokyo, Japan; Institut Jacques Monod, France

## Abstract

**Background:**

Although *TBX1* mutations have been identified in patients with 22q11.2 deletion syndrome (22q11.2DS)-like phenotypes including characteristic craniofacial features, cardiovascular anomalies, hypoparathyroidism, and thymic hypoplasia, the frequency of *TBX1* mutations remains rare in deletion-negative patients. Thus, it would be reasonable to perform a comprehensive genetic analysis in deletion-negative patients with 22q11.2DS-like phenotypes.

**Methodology/Principal Findings:**

We studied three subjects with craniofacial features and hypocalcemia (group 1), two subjects with craniofacial features alone (group 2), and three subjects with normal phenotype within a single Japanese family. Fluorescence *in situ* hybridization analysis excluded chromosome 22q11.2 deletion, and genomewide array comparative genomic hybridization analysis revealed no copy number change specific to group 1 or groups 1+2. However, exome sequencing identified a heterozygous *TBX1* frameshift mutation (c.1253delA, p.Y418fsX459) specific to groups 1+2, as well as six missense variants and two in-frame microdeletions specific to groups 1+2 and two missense variants specific to group 1. The *TBX1* mutation resided at exon 9C and was predicted to produce a non-functional truncated protein missing the nuclear localization signal and most of the transactivation domain.

**Conclusions/Significance:**

Clinical features in groups 1+2 are well explained by the *TBX1* mutation, while the clinical effects of the remaining variants are largely unknown. Thus, the results exemplify the usefulness of exome sequencing in the identification of disease-causing mutations in familial disorders. Furthermore, the results, in conjunction with the previous data, imply that *TBX1* isoform C is the biologically essential variant and that *TBX1* mutations are associated with a wide phenotypic spectrum, including most of 22q11.2DS phenotypes.

## Introduction

Chromosome 22q11.2 deletion syndrome (22q11.2DS) is a developmental disorder associated with characteristic craniofacial features with velopharyngeal incompetence, cardiovascular anomalies primarily affecting the outflow tracts, hypoparathyroidism and resultant hypocalcemia, and thymic hypoplasia leading to susceptibility to infection [1]. This condition is also frequently accompanied by non-specific clinical features such as developmental retardation [1]. Expressivity and penetrance of these features are highly variable and, consistent with this, chromosome 22q11.2 deletions have been identified in DiGeorge syndrome (DGS) and velocardiofacial syndrome (VCFS) with overlapping but different patterns of clinical features [1].

While multiple genes are involved in chromosome 22q11.2 deletions [2], *TBX1* (T-box 1) has been regarded as the major gene relevant to the development of clinical features in 22q11.2DS [3]. Indeed, heterozygous *TBX1* mutations have been identified in several deletion-negative patients with 22q11.2DS phenotype [2]–[8], and mouse studies argue for the critical role of *Tbx1* in the development of 22q11.2DS phenotypes [3]. However, the frequency of *TBX1* mutations remains rare in deletion-negative patients: Gong et al. identified only a few probable *TBX1* mutations after studying 40 patients with DGS/VCFS phenotypes [4], and Zweier et al. found a single *TBX1* mutation after examining 10 patients with 22q11.2DS phenotype [8]. This indicates the presence of genetic heterogeneity in the development of 22q11.2DS phenotype in deletion-negative patients. Consistent with this, another DGS/VCFS locus has been assigned to chromosome 10p13-14 region [9]. Thus, it would be reasonable to perform a comprehensive genetic analysis in deletion-negative patients with 22q11.2DS phenotype.

In this regard, recent advance in molecular technologies has enabled to perform comprehensive genetic analyses, thereby contributing to the identification of underlying factors in genetic disorders. Indeed, genomewide array comparative genomic hybridization (CGH) has identified multiple disease-associated copy-number changes [10], and exome sequencing has discovered multiple disease-causing gene mutations [11]. In particular, these technologies can be powerful methods for familial disorders, because it is predicted that a single copy-number change or mutation is shared in common by affected subjects and is absent from non-affected subjects within a family.

Here, we performed array CGH analysis and exome sequencing in a family with 22q11.2DS-like clinical features. Although this study did not discover a novel disease gene, a *TBX1* mutation was successfully identified.

## Materials and Methods

### Ethics statement

The Institutional Review Board Committees of Hamamatsu University School of Medicine, Tohoku University School of Medicine, Kurashiki Central Hospital, and National Research Institute for Child Health and Development considered and approved the study, consent/assent procedures, and the publication of images and case details associated with this work. The individuals in this manuscript have given written informed consent (as outlined in PLOS consent form) to publish these case details. Actually, this study was performed after obtaining written informed consent from the parents of the child subjects and from the adult subjects. Furthermore, the mother and the elder brother aged 19 years old have given written informed consent to publication of the facial photographs of the two brothers; in addition, the younger brother aged 10 years has given informed assent.

### Clinical Report

**Figure 1:**
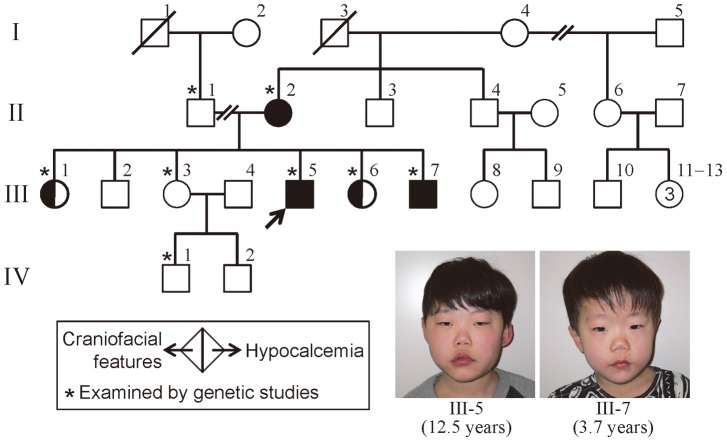
The pedigree of this family. Facial features of subjects III-5 and III-7 are shown.

The pedigree of this Japanese family is shown in Fig. 1, and clinical findings of the family members are summarized in Table 1. The proband (subject III-5) was found to have hypocalcemia and hyperphosphatemia in a pre-operation laboratory test for repeated otitis media at 8 years of age, and was referred to Department of Pediatrics at Kurashiki Central Hospital. Subsequent examination revealed borderline low serum intact PTH value. Thus, he was diagnosed as having hypoparathyroidism, and received vitamin D therapy. Furthermore, physical examination showed characteristic craniofacial features with velopharyngeal incompetence suggestive of 22q11.2DS.

**Table 1:** Clinical findings of the family members.

	*TBX1* mutation (+)					*TBX1* mutation (−)			*TBX1* mutation (N.E.)		
Individual	II-2	III-1	III-5	III-6	III-7	II-1	III-3	IV-1	II-3	II-4	III-2
Present age (year)	51	26	19	13	10	59	22	5	50	49	25
Sex	F	F	M	F	M	M	F	M	M	M	M
Craniofacial features	+	+	+	+	+	−	−	−	−	−	−
Hypertelorism	+	+	+	+	+	−	−	−	−	−	−
Blepharophimosis	+	+	+	+	+	−	−	−	−	−	−
Low set ears	+	+	+	+	+	−	−	−	−	−	−
Auricular anomalies	+	−	−	−	−	−	−	−	−	−	−
Narrow nose	+	+	+	+	+	−	−	−	−	−	−
Cleft palate	−	−	−	−	−	−	−	−	−	−	−
Micrognathia	±	+	+	+	+	−	−	−	−	−	−
Velopharyngeal incompetence	+^d^	+	+	+	+	−	−	−	−	−	−
Hypoparathyroidism	+	−	+	−	+	−	−	−	−	−	−
Age at examination (year)	44	17	8	4	0 (1 day)	N.E.	15	0 (6 days)	N.E.	N.E.	18
Serum calcium (mg/dL)^a^	7.6^e^	9.0	6.0	9.1	5.9	…	9.0	9.8	…	…	9.6
Serum i-phosphate (mg/dL)^a^	3.9^e^	4.9	9.1	5.0	N.E.	…	4.8	6.3	…	…	4.6
Serum intact PTH (pg/dL)^a^	31^e^	N.E.	15	N.E.	19	…	N.E.	34	…	…	N.E.
Cardiovascular anomalies^b^	−	−	−	−	−	−	−	−	−	−	−
Hypoplastic thymus^c^	−	N.E.	N.E.	N.E.	N.E.	N.E.	N.E.	N.E.	N.E.	N.E.	N.E.
Susceptible to infection	−	−	−f	−	−	−	−	−	−	−	−
Other features	−	−	−	−	−	−	−	−	−	−	−
Developmental retardation	+	+	+^g^	+	+^g^	−	−	−	−	−	−
Sensorineural deafness	+^h^	−	−	−	−	−	−	−	−	−	−
Graves' disease	−	−	+^i^	−	−	−	−	−	−	−	−

Individuals correspond to those shown in Fig. 1.

i-phosphate: inorganic phosphate; SD: standard deviation; F: female; M: male; and N.E.: not examined.

a Reference values: calcium, 9.0–11.0 mg/dL in infants and 8.8–10.2 mg/dL in adults; inorganic phosphate, 4.8–7.5 mg/dL in infants and 2.5–4.5 mg/dL in adults, and intact PTH, 10–65 pg/dL in infants and 14–55 pg/dL in adults.

Conversion factor to the SI unit: 0.25 for calcium (mmol/L), 0.32 for inorganic phosphate (mmol/L), and 0.106 for intact PTH (pmol/L).

b Examined by echocardiography, chest roentgenography, and/or electrocardiography.

c Examined by computed tomography.

d Received velopharyngeal closure.

e On treatment with vitamin D.

f Repeated otitis media only.

g Received speech therapy.

h Required hearing aids.

i At the time of diagnosis (11 years of age), serum TSH was <0.01 mIU/L, free T_3_ 33.1 pg/mL [51.0 pmol/L], free T_4_ 5.11 ng/dL [65.8 nmol/L], and TSH receptor antibody 1284% [normal range <1.9%].

Similar craniofacial features were also exhibited by subjects II-2, III-1, III-6, and III-7, and hypocalcemia was also identified in subjects II-2 and III-7. Actually, subject II-2 was taking vitamin D, and subject III-7 was noticed to have hypocalcemia at birth because of the history of subject III-5, and was treated with vitamin D. The five subjects with 22q11.2DS-like craniofacial features lacked cardiovascular anomalies; while they also lacked susceptibility to infection, except for repeated otitis media in subject III-5, thymic hypoplasia was not evaluated in four of the five subjects. By contrast, the five subjects exhibited borderline to mild developmental delay. Indeed, adult subjects II-2 and III-1 had some difficulty in verbal communications, although they were able to get on their daily life, and subject II-2 was able to take care of family members. Similarly, child subjects III-5, III-6, and III-7 also showed speech delay, and subjects III-5 and III-7 received speech therapy. Furthermore, subject III-7 was diagnosed as having pervasive developmental disorder, and his verbal, performance, and full scale intelligence quotients were assessed as 63, 64, and 60, respectively, by the WISC-III method at 10 years of age. In addition, subject II-2 had sensorineural deafness, and subject III-5 had Graves' disease.

### Molecularly Studied Subjects

Molecular studies were performed for eight subjects in this family, using peripheral blood samples. They were divided into three groups in terms of clinical findings: group 1, subjects II-2, III-5, and III-7 with craniofacial features and hypocalcemia; group 2, subjects III-1 and III-6 with craniofacial features alone; and group 3, subjects II-1, III-3, and IV-1 with apparently normal phenotype (Fig. 1).

### FISH and Array CGH Analyses

Fluorescence *in situ* hybridization (FISH) analysis was performed with a probe for *HIRA* on the commonly deleted chromosome 22q11.2 region and that for *ARSA* at chromosome 22q13 utilized as an internal control (Abott). Array CGH was carried out using a genomewide 2x400K Agilent platform catalog array, according to the manufacturer's instructions (Agilent Technologies), and copy number variants/polymorphisms were screened with Agilent Genomic Workbench software using the Database of Genomic Variants (http://dgv.tcag.ca/dgv/app/home).

### Exome and Sanger Sequencings

Exon capture was performed with the SureSelect Human All Exon kit v4 (Agilent Technologies). Exon libraries were sequenced with the Illumina Hiseq 2000 platform according to the manufacturer's instructions (Illumina), providing 108–122 average depth for each sample. Paired 101-base pair reads were aligned to the reference human genome (UCSChg19) using the Burrows-Wheeler Alignment tool [12]. Likely PCR duplicates were removed with the Picard program (http://picard.sourceforge.net/). Single-nucleotide variants and indels were identified using the Genome Analysis Tool Kit (GATK) v1.6 software [13]. SNVs and indels were annotated against the RefSeq database, 1000 Genomes Project variant data, and dbSNP135 with the ANNOVAR program [14].

To confirm mutations indicated by exome sequencing, Sanger sequencing was performed for PCR products obtained with primers flanking the detected mutations, using a 3500xL genetic analyzer (Life Technologies). Furthermore, the PCR products were subcloned with TOPO TA Cloning Kit (Life Technologies), and normal and mutant alleles were sequenced separately.

### *In silico* protein functional analysis

Function of proteins with missense variations was assessed by Polymorphism Phenotyping-2 (PolyPhen-2, http://genetics.bwh.harvard.edu/pph2/) and Sorting Intolerant From Tolerant (SIFT, http://sift.jcvi.org/), and that of proteins with in-frame amino acid deletions was evaluated by PROVEAN predictions (http://provean.jcvi.org/index.php).

## Results

### FISH and Array CGH Analyses

**Figure 2:**
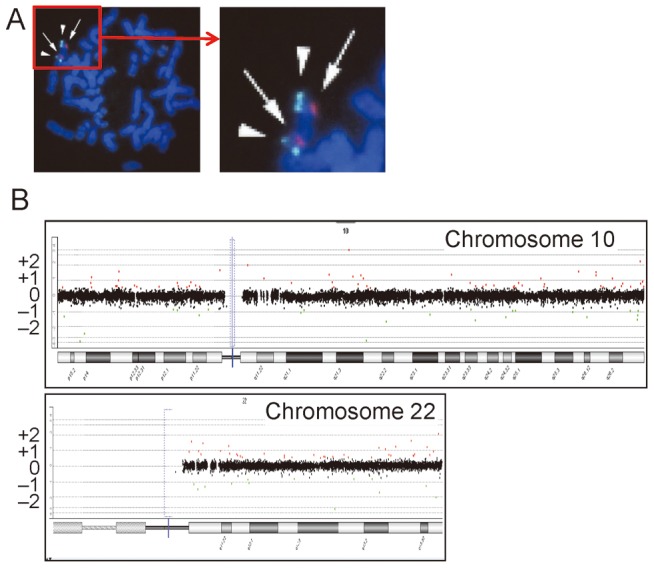
FISH and array CGH analyses in the proband (III-5). **A.** FISH analysis. Two signals are shown for both *HIRA* at 22q11.2 (red signals indicated by arrows) and *ARSA* at 22q13 (green signals indicated by arrowheads). **B.** Array CGH analysis. No copy number change is found for chromosome 10 carrying the second DiGeorge region and chromosome 22 harboring the DGS/VCFS critical region, as well as other chromosomes (not shown). Black, red, and green dots denote signals indicative of the normal, the increased (>+0.5), and the decreased (<−0.8) copy numbers, respectively. Although several red and green signals are seen, there is no portion associated with ≥3 consecutive red or green signals.

FISH analysis delineated two signals for *HIRA* (Fig. 2A). Array CGH analysis revealed no copy number change specific to group 1 or groups 1+2, in the entire genome including chromosome 10p13-14 and chromosome 22q11.2 regions (Fig. 2B).

### Exome and Sanger Sequencings

**Figure 3:**
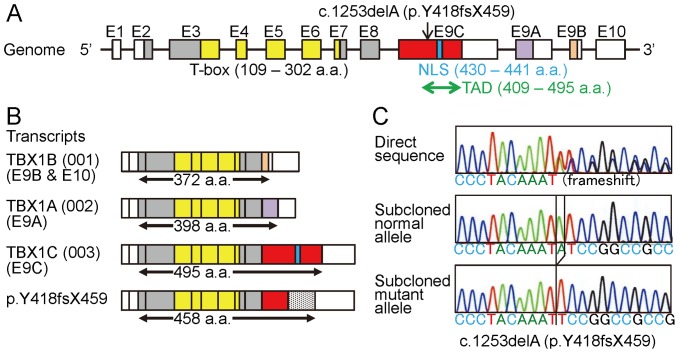
*TBX1* mutation identified in this family. **A.** Genomic structure of *TBX1* and the position of the mutation. The color and the white boxes represent the coding regions and the untranslated regions on exons 1–10 (E1–E10), respectively; the red, the purple, and the orange segments indicate the coding regions on the final exons 9C, 9A, and 9B (splice variants), respectively. The T-box is indicated by yellow boxes, the nuclear localization signal (NLA) by a blue segment, and the transactivation domain (TAD) by a green arrow. The c.1253delA (p.Y418fsX459) identified in this family resides on exon 9C. **B.** Transcripts of *TBX1*. Three variants are formed by alternative splicing of the final exons 9C, 9A, and 9B. The c.1253delA (p.Y418fsX459) mutation is predicted to yield a truncated TBX1C protein missing the NLS and most of the TAD. The stippled box of p.Y418fsX459 denotes aberrant amino acid sequence produced by the frameshift mutation. **C.** Electrochromatograms showing the frameshift mutation by Sanger sequencing. The primer sequences used are: 5′-GCGGCCAAGAGCCTTCTCT-3′ and 5′-GGGTGGTAGCCGTGGCCA-3′.

Exome sequencing identified nine heterozygous non-synonymous variants (six missense variants, two in-frame microdeletions, and one frameshift variant) that were specific to groups 1+2 (namely, they were present in groups 1+2 and absent from group 3 as well as from 1000 Genomes, dbSNP135, and our in-house exome data from 70 individuals) (Table S1). Notably, the frameshift variant (c.1253delA, p.Y418fsX459) was found at exon 9C of *TBX1* for DGS/VCFS (Fig. 3). Of the remaining eight variants, two variants were also detected in disease-related genes: p.G204R in *HDAC4* for brachydactyly-mental retardation syndrome [15], and p.276del in *CCND1* constituting a susceptibility factor for colorectal cancer and a modifier for von Hippel-Lindau disease [16], [17]. Exome sequencing also detected two heterozygous missense variants that were specific to group 1 (Table S1).

When all variants were included, exome sequencing revealed: (1) 83 non-synonymous and 86 synonymous variants that were present in groups 1+2 and absent from group 3 (Table S2); (2) 54 non-synonymous and 48 synonymous variants that were present in group 1 and absent from groups 2+3 (Table S3); (3) 6,033 non-synonymous and 6,667 synonymous variants that were present in groups 1+2, but not specific to groups 1+2 (thus, they may be present in group 3 or absent from group 3); and (4) 7,073 non-synonymous and 7,861 synonymous variants that were present in group 1, but not specific to group 1. Furthermore, comparison of the exome sequencing data between group 1 with hypocalcemia and group 2 without hypocalcemia revealed 231 non-synonymous and 254 synonymous variants that were present in group 1 and absent from group 2 (Table S4), and 246 non-synonymous and 242 synonymous variants that were present in group 2 and absent from group 1 (Table S5). (The variant data other than those described in Supplemental Tables may be available on request after discussion with the family members and approval by our IRBs, because they contain a huge amount of individual genetic information.)

### *In silico* protein functional analysis

The results are summarized in Table S1. The p.G204R in *HDAC4* and the p.E276del in *CCND1* were assessed as non-pathologic, while some variants were evaluated as potentially pathologic.

## Discussion

Exome sequencing successfully identified a heterozygous frameshift variant on exon 9C of *TBX1*. The c.1253delA (p.Y418fsX459) appears to be a disease-causing mutation, because it is predicted that this variant escapes nonsense-mediated mRNA decay due to its position on the final exon [18] and produces a truncated protein lacking the nuclear localization signal (NLS) and most of the transactivation domain (TAD) on exon 9C (Fig. 3) [19]. In support of this, functional studies for a similar c.1223delC (p.S408fsX459) mutation on exon 9C have shown that the truncated p.S408fsX459 protein was incapable of localizing to nucleus and lost transactivation function [2], [5], [19]. One may argue that this c.1253delA mutation affects *TBX1* isoform C (*TBX1C*, TBX1-003) alone, while *TBX1* produces three transcript variants containing T-box [2], [4] (Fig. 3). However, *TBX1C* is the major transcript with the NLS and the TAD in human and is highly homologous to mouse *Tbx1* [4] (Fig. S1).

Craniofacial features in groups 1+2 and hypocalcemia in group 1 are well explained by the *TBX1* mutation [3]. This argues for a critical role of this mutation in the phenotypic development in groups 1+2, while the clinical effects of the remaining variants identified by exome sequencing are largely unknown. In this regard, comparison between group 1 with hypocalcemia and group 2 without hypocalcemia revealed a large number of non-synonymous and synonymous variants that exclusively belonged to either group 1 (Table S4) or group 2 (Table S5), although the lists did not contain a c.2968A>G (p.R990G) SNP in *CASR* (calcium sensing receptor) that has a gain-of-function effect and appears to raise the susceptibility to hypocalcemia (Fig. S2) [20]. Thus, it is likely that, together with environmental factors, the combination of hitherto unknown calcium metabolism-related functional variants would underlie different serum calcium values between groups 1 and 2.

In addition to craniofacial features with and without hypocalcemia, *TBX1* mutation positive subject II-2 had sensorineural deafness, and III-5 had Graves' disease. Since such features are occasionally manifested by patients with 22q11.2DS [21], [22], the results may suggest the relevance of *TBX1* to such rather infrequent features in 22q11.2DS.

The five *TBX1* mutation positive subjects in groups 1+2 lacked cardiovascular lesion and manifested borderline to mild developmental retardation (while they had no susceptibility to infection, assessment of thymic hypoplasia remained fragmentary). By contrast, cardiovascular lesion is frequently observed and developmental retardation is rare in previously reported patients with *TBX1* mutations, although clinical features are fairly variable among mutation positive patients (Table 2). Such difference would more or less be ascribed to an examination bias that *TBX1* has been analyzed in patients with isolated cardiovascular lesion in several studies [4], [6], [7] or to the functional difference of the mutant proteins [2], [5]–[8]. However, in seven patients who have been examined for DGS/VCFS-like clinical features and found to have frameshift mutations on exon 9C (p.S408fsX459, p.H425fsX613, and p.S431fsX608) affecting the NLS and the TAD, cardiovascular lesion was present in four patients and developmental delay was absent or not described, despite apparent similarity in the ascertainment of patients and the function of mutant proteins between the seven patients and the five affected subjects in this family (Table 2) [2]–[5].

**Table 2:** Summary of patients with *TBX1* mutations.

	TBX1C only					TBX1A–C				22q11.2DS
Position	Exon 9C	Exon 9C	Exon 9C	Exon 9C	Exon 9C	Exon 3	Exon 4	Exon 5	Exon 8	
cDNA change^a^	c.1223	c.1253	c.1274_1281	c.1293_1315	c.1399_1428	c.129_185	c.443T>A	c.582C>G	c.928G>A	Deletion
	delC	delA	del8	del23^h^	dup30^k^	del57^k^				
Amino acid	p.S408	p.Y418	p.H425	p.S431	p.467_476	p.43_61	p.F148Y	p.H194Q	p.G310S	
change	fsX459	fsX459	fsX613	fsX608	dup10A	del19				
NLS (exon 9C)	−	−	−	+^i^	+^l^	+	+	+	+	
TAD (exon 9C)	−	Involved	Involved	Involved	Involved	+	+	+	+	
Function	LOF	N.E.	N.E.	LOF	LOF	Reduced	GOF^m^	GOF^m^	GOF^m^	
Patient number	3	5	1	3^j^	2	1	1	2	1	558
Occurrence	Familial	Familial	Sporadic^g^	Familial	Sporadic	Sporadic^g^	Sporadic	Familial	Sporadic	
Facial features^b^	3/3	5/5	+	3/3	0/2	−	+	2/2	+	100%
Nasal voice^c^	2/3	5/5	N.D.	3/3	0/2	−	+	0/2	+	32%
Cardiovascular	1/3	0/5	+	2/3	2/2	+	+	0/2	+	57%
anomalies										
Hypopara-	1/3	3/5	+	N.D.	0/2	−	−	0/2	+	60%
thyroidism^d^										
Hypoplastic	1/2^e^	0/1^f^	+	N.D.	0/2	−	−	N.E.	+	?
thymus										
Susceptible to	N.D.	0/5	N.D.	N.D.	0/2	−	N.D.	N.D.	N.D.	?
infection										
Developmental	0/3	5/5	N.D	0/3	0/2	−	−	1/2	−	38%
retardation										
Reference	2	This study	3, 4	5	4, 6	7	2	8	2	1

In addition to the mutations listed in this table, several missense variants and in-frame indels with unknown functions have been found in patients with isolated cardiovascular anomalies and in those with DGS/VCFS-like phenotype [4].

NLS: nuclear localization signal; TAD: transactivation domain; LOF: loss-of-function; N.D.: not described; N.E.: not examined; GOF: gain-of-function; Del: deletion; and Dup: duplication.

a According to NM_080647.

b Suggestive of 22q11.2 deletion syndrome.

c Velopharygeal insufficiency.

d Hypocalcemia is included.

e Two of the three subjects have been examined for hypoplastic thymus.

f One of the five subjects has been examined for hypoplastic thymus.

g These two mutations have been inherited from apparently normal mothers.

h The c.1293-1315del23 has been described as c.1320-1342del23 in the original report [5].

i Although the natural NLS has been disrupted, a new NLS-compatible motif (RGRRRRCR) has been created on the added amino acid sequence.

j Another deceased individual in this family also has similar clinical features.

k These two mutations have been identified in *TBX1* analyses for patients with cardiovascular anomalies only.

l The mutant protein is aggregated in the cytoplasm and the nucleus.

m Gain-of-function effects have been found by *in vitro* studies [8].

Thus, there may be protective factor(s) for cardiovascular lesion and susceptibility factor(s) for developmental delay in groups 1+2. In this regard, a simple explanation would be that protective factor(s) for cardiovascular lesion are present in groups 1+2 and may be present in group 3 or absent from group 3, whereas susceptibility factor(s) for developmental delay is present in groups 1+2 and absent from group 3. Since 6,033 non-synonymous and 6,667 synonymous variants were found to be present in groups 1+2 but not specific to groups 1+2, and 83 non-synonymous and 86 synonymous variants were revealed to be present in groups 1+2 and absent from group 3, a certain fraction of functional variants may constitute protective factor(s) for cardiovascular lesion and susceptibility factor(s) for developmental delay. In addition, while p.G204R on *HDAC4* for brachydactyly-mental retardation syndrome was assessed as non-pathologic by *in silico* analysis, it may have played a certain role in the occurrence of developmental delay in groups 1+2. Actually, such protective and susceptibility factor(s) would be more complex, with the effects of functional variants unique to each patient as well as the influences of environmental factors. Furthermore, it remains possible that the c.1253delA (p.Y418fsX459) mutation found in this study may be related to a specific phenotype characterized by the presence of craniofacial features and developmental delay and by the absence of cardiovascular lesion, because of a hitherto unrevealed mechanism(s). This matter awaits further studies.

Besides the clinical findings, several matters are also notable in the nine apparently pathologic *TBX1* mutations identified to date (Table 2). First, the mutations reside on exons 3–8 common to isoforms A–C or on exon 9C specific to isoform C, with no mutation on exons 9A and 9B specific to isoforms A and B. This would be consistent with *TBX1C* having the primary biological function. Second, while most mutations have loss-of-function effects, gain-of-function effects have been suggested for p.F148Y, p.H194Q, and p.310S by *in vitro* studies [8]. Thus, *TBX1* loss-of-function mutations and gain-of-function mutations may result in overlapping clinical features. Lastly, the c.1274_1281delACTATCTC (p.H425fsX613) missing the NLS on exon 9C was shared by a patient with DGS-like phenotype and the apparently normal mother, and the c.129_185del57 (p.43-61del19) with reduced transcriptional activity was common to a patient with non-syndromic tetralogy of Fallot and the apparently normal mother. This would imply the reduced penetrance of phenotypes caused by these mutations.

In summary, we identified a *TBX1* mutation by exome sequencing in a family with chromosome 22q11.2 deletion-like phenotype. Application of such powerful methods will serve to identify a causative gene in genetically heterogeneous disorders.

## Supporting Information

Figure S1 **Comparison of amino acid sequence of human** ***TBX1C*** **and mouse** ***Tbx1*** **.** The T-box is highlighted in yellow, and the nuclear localization signal in light blue. The region for transactivation domain is surrounded by squares. The Y highlighted in red denotes the amino acid residue where the frameshift mutation in this family has taken place.
(TIF)

Figure S2 **Analysis of c.2968A>G SNP (p.R990G, rs1042636) with a gain-of-function effect in exon 7 of** ***CASR*** **.** The SNP pattern is not co-segregated with the presence or absence of hypocalcemia.
(TIF)

Table S1 **Summary of heterozygous non-synonymous variants.**
(PDF)

Table S2 **A list of variants that are present in groups 1+2 and absent from group 3.**
(PDF)

Table S3 **A list of variants that are present in group 1 and absent from groups 2+3.**
(PDF)

Table S4 **A list of variants that are present in group 1 and absent from group 2.**
(PDF)

Table S5 **A list of variants that are present in group 2 and absent from group 1.**
(PDF)

## References

[1] Ryan AK, Goodship JA, Wilson DI, Philip N, Levy A, et al (1997) Spectrum of clinical features associated with interstitial chromosome 22q11 deletions: a European collaborative study. J Med Genet 34: 798–804. 9350810 10.1136/jmg.34.10.798 PMC1051084

[2] Yagi H, Furutani Y, Hamada H, Sasaki T, Asakawa S, et al (2003) Role of TBX1 in human del22q11.2 syndrome. Lancet 362: 1366–1373. 14585638 10.1016/s0140-6736(03)14632-6

[3] Baldini A (2005) Dissecting contiguous gene defects: TBX1. Curr Opin Genet Dev 15: 279–84. 15917203 10.1016/j.gde.2005.03.001

[4] Gong W, Gottlieb S, Collins J, Blescia A, Dietz H, et al (2001) Mutation analysis of TBX1 in non-deleted patients with features of DGS/VCFS or isolated cardiovascular defects. J Med Genet 38: E45. 11748311 10.1136/jmg.38.12.e45 PMC1734783

[5] Paylor R, Glaser B, Mupo A, Ataliotis P, Spencer C, et al (2006) Tbx1 haploinsufficiency is linked to behavioral disorders in mice and humans: implications for 22q11 deletion syndrome. Proc Natl Acad Sci U S A 103: 7729–7734. 16684884 10.1073/pnas.0600206103 PMC1472513

[6] Rauch R, Hofbeck M, Zweier C, Koch A, Zink S, et al (2010) Comprehensive genotype-phenotype analysis in 230 patients with tetralogy of Fallot. J Med Genet 47: 321–331. 19948535 10.1136/jmg.2009.070391

[7] Griffin HR, Töpf A, Glen E, Zweier C, Stuart AG, et al (2010) Systematic survey of variants in TBX1 in non-syndromic tetralogy of Fallot identifies a novel 57 base pair deletion that reduces transcriptional activity but finds no evidence for association with common variants. Heart 96: 1651–1655. 20937753 10.1136/hrt.2010.200121 PMC2976076

[8] Zweier C, Sticht H, Aydin-Yaylagül I, Campbell CE, Rauch A (2007) Human TBX1 missense mutations cause gain of function resulting in the same phenotype as 22q11.2 deletions. Am J Hum Genet 80: 510–517. 17273972 10.1086/511993 PMC1821102

[9] Daw SCM, Taylor C, Kraman M, Call K, Mao J, et al (1996) A common region of 10p deleted in DiGeorge and velocardiofacial syndromes. Nat Genet 13: 458–461. 8696341 10.1038/ng0896-458

[10] McDonnell SK, Riska SM, Klee EW, Thorland EC, Kay NE, et al (2013) Experimental designs for array comparative genomic hybridization technology. Cytogenet Genome Res 139: 250–257. 23548696 10.1159/000348815 PMC3728659

[11] Wang Z, Liu X, Yang B-Z, Gelernter J (2013) The role and challenges of exome sequencing in studies of human diseases. Front Genet doi: 10.3389/fgene.2013.00160 10.3389/fgene.2013.00160 PMC3752524 24032039

[12] Li H, Durbin R (2009) Fast and accurate short read alignment with Burrows-Wheeler transform. Bioinformatics 25: 1754–1760. 19451168 10.1093/bioinformatics/btp324 PMC2705234

[13] McKenna A, Hanna M, Banks E, Sivachenko A, Cibulskis K, et al (2010) The Genome Analysis Toolkit: a MapReduce framework for analyzing next-generation DNA sequencing data. Genome Res 20: 1297–1303. 20644199 10.1101/gr.107524.110 PMC2928508

[14] Wang K, Li M, Hakonarson H (2010) ANNOVAR: functional annotation of genetic variants from high-throughput sequencing data. Nucleic Acids Res 38: e164. 20601685 10.1093/nar/gkq603 PMC2938201

[15] Williams SR, Aldred MA, Der Kaloustian VM, Halal F, Gowans G, et al (2010) Haploinsufficiency of HDAC4 causes brachydactyly mental retardation syndrome, with brachydactyly type E, developmental delays, and behavioral problems. Am J Hum Genet 87: 219–228. 20691407 10.1016/j.ajhg.2010.07.011 PMC2917703

[16] Kong S, Amos CI, Luthra R, Lynch PM, Levin B, et al (2000) Effects of cyclin D1 polymorphism on age of onset of hereditary nonpolyposis colorectal cancer. Cancer Res 60: 249–252. 10667569

[17] Zatyka M, da Silva NF, Clifford SC, Morris MR, Wiesener MS, et al (2002) Identification of cyclin D1 and other novel targets for the von Hippel-Lindau tumor suppressor gene by expression array analysis and investigation of cyclin D1 genotype as a modifier in von Hippel-Lindau disease. Cancer Res 62: 3803–3811. 12097293

[18] Holbrook JA, Neu-Yilik G, Hentze MW, Kulozik AE (2004) Nonsense-mediated decay approaches the clinic. Nat Genet 36: 801–808. 15284851 10.1038/ng1403

[19] Stoller JZ, Epstein JA (2005) Identification of a novel nuclear localization signal in Tbx1 that is deleted in DiGeorge syndrome patients harboring the 1223delC mutation. Hum Mol Genet 14: 885–892. 15703190 10.1093/hmg/ddi081

[20] Vezzoli G, Terranegra A, Arcidiacono T, Biasion R, Coviello D, et al (2007) R990G polymorphism of calcium-sensing receptor does produce a gain-of-function and predispose to primary hypercalciuria. Kidney Int 71: 1155–1162. 17332735 10.1038/sj.ki.5002156

[21] Ohtani I, Schuknecht HF (1984) Temporal bone pathology in DiGeorge's syndrome. Ann Otol Rhinol Laryngol 93 3 Pt 1: 220–224. 6732106 10.1177/000348948409300306

[22] Kawame H, Adachi M, Tachibana K, Kurosawa K, Ito F, et al (2001) Graves' disease in patients with 22q11.2 deletion. J Pediatr 139: 892–895. 11743521 10.1067/mpd.2001.119448

